# Recombination in Hepatitis C Virus

**DOI:** 10.3390/v3102006

**Published:** 2011-10-24

**Authors:** Fernando González-Candelas, F. Xavier López-Labrador, María Alma Bracho

**Affiliations:** Unidad Mixta “Genómica y Salud” CSISP, Instituto Cavanilles de Biodiversidad y Biología Evolutiva, Universidad de Valencia, Catedrático José Beltrán 2, E-46980 Paterna, Valencia, Spain; E-Mails: f.xavier.lopez@uv.es (F.X.L.-L.); bracho_alm@gva.es (M.A.B.)

**Keywords:** phylogenetic tree, congruence, breakpoint, superinfection, homoplasy

## Abstract

Hepatitis C virus (HCV) is a *Flavivirus* with a positive-sense, single-stranded RNA genome of about 9,600 nucleotides. It is a major cause of liver disease, infecting almost 200 million people all over the world. Similarly to most RNA viruses, HCV displays very high levels of genetic diversity which have been used to differentiate six major genotypes and about 80 subtypes. Although the different genotypes and subtypes share basic biological and pathogenic features they differ in clinical outcomes, response to treatment and epidemiology. The first HCV recombinant strain, in which different genome segments derived from parentals of different genotypes, was described in St. Petersburg (Russia) in 2002. Since then, there have been only a few more than a dozen reports including descriptions of HCV recombinants at all levels: between genotypes, between subtypes of the same genotype and even between strains of the same subtype. Here, we review the literature considering the reasons underlying the difficulties for unequivocally establishing recombination in this virus along with the analytical methods necessary to do it. Finally, we analyze the potential consequences, especially in clinical practice, of HCV recombination in light of the coming new therapeutic approaches against this virus.

## 1. Introduction

Hepatitis C virus (HCV) infects about 170 million people worldwide, about 3% of the world’s population [1]. Infection by HCV is the major cause of liver disease and a potential cause of substantial morbidity and mortality in the future [2]. Until the recent discovery of a canine relative [3], HCV was the only species of the genus *Hepacivirus* within the family *Flaviviridae*. It has a single stranded, positive-sense, non-segmented RNA genome of about 9,600 nucleotides (nt) and a single, long open reading frame encoding a polyprotein of about 3,000 amino acids with the gene order C-E1-E2-p7-NS2-NS3-NS4A-NS4B-NS5A-NS5B. In addition, alternative translation products (F protein) have been detected from a reading frame overlapping the core gene (core+1/ARFP) [4], but its biological role remains elusive. The structural proteins are C (core) and E1 and E2 (envelope glycoproteins). The p7 protein seems to be an ion channel [5]. The NS2 through NS5 regions encode the remaining non-structural proteins [6]; NS2 is a transmembrane protein; NS3 has a dual role as serine protease and RNA helicase; NS4A is a cofactor of NS3; NS4B is a membrane-associated protein; NS5A is a component of the replicase complex, and NS5B is the RNA-dependent RNA polymerase.

Six major genotypes and about 80 subtypes of HCV have been described [7,8] based on levels of sequence divergence. HCV genotypes have been shown to be distributed over distinct geographical areas and although they share their most basic biological features, there seems to be some differences in their susceptibility to interferon (IFN)-based therapies [9,10]. Genotype 1 is the predominant variant in developed countries and shows the poorest response to therapy. Although with a lower frequency, infections with HCV genotypes 2 and 3 are also common in Europe, and show the best response rates to IFN-based therapies.

HCV is mainly transmitted by parenteral routes and differences in their transmission rates can be an important factor to explain the differences in prevalence of a genotype/subtype in different geographic regions [11–13]. Needle sharing among intravenous drug users (IDUs) currently represents the most common route of acquisition of HCV in the developed world [14]. Because HCV and human immunodeficiency virus (HIV) share blood-borne transmission routes, HIV/HCV co-infection is relatively frequent, especially in countries such as Spain and Italy, with a high prevalence of HCV/HIV infection due to injecting drug use [15].

Like HIV-1, HCV is characterized by high levels of genetic heterogeneity [16,17] which impact heavily on different aspects such as viral persistence, susceptibility to treatment, and disease progression, and lack of an efficient vaccine, among others [18–20]. As for other RNA viruses, mutation at the nucleotide level seems to be the primary cause of genetic variation in HCV, due to the use of an error-prone, non-proofreading RNA-dependent RNA-polymerase for replication of its genetic material. The mutation rate of HCV, estimated at 10^−4^ substitutions per site and round of copying [21], is among the highest for RNA viruses including retroviruses [22], and would seem to be high enough to generate all the genetic variation found in this virus. Alternative mechanisms for generating genetic variability in viruses, such as reassortment, cannot operate in HCV, because it has a non-segmented genome, or act very sparingly, such as the introduction of indels [23].

An additional mechanism generating genetic variation in viruses is recombination. Although recombination is usually important in most RNA viruses, until recently the evidence for recombination in HCV was scarce [24], suggesting that these events are rare *in vivo* and that the resulting recombinants are usually not viable [25]. However, in the last few years, a few natural inter-genotype, intra-genotype (inter-subtype) and even intra-strain recombinants of HCV have been identified, changing previous ideas about the absence of recombination in this virus. This view was based on two main reasons. On the one hand, until 2002 not a single, convincing case of a well characterized HCV recombinant had been identified [26]. Additionally, experimental evidence suggested that superinfection by another HCV isolate was prevented in HCV-infected cells [27]. Given that for viral recombination to occur it is necessary that two different viruses infect the same cell simultaneously, the impossibility of superinfection also prevented the appearance of recombinant viruses.

Here, we review the evidence for recombination in HCV and analyze why it is so difficult to detect it. Additionally, we consider how to obtain convincing evidence of recombination and its possible consequences, both at the evolutionary and clinical levels.

## 2. Reports of Recombination in HCV

The first convincing report of an HCV recombinant strain was published in 2002 by Kalinina *et al.* [26]. Some earlier reports had already described “chimeric” strains in which partial sequences for different regions in the viral genome led to discordant genotyping of two isolates from Honduras [24], with the 5′-end (at least portions of the core and E2 and p7 genes) corresponding to subtype 1a and the 3′-end (NS5 gene) to subtype 3a. As no further sequencing was possible for these isolates, it was not possible to determine their exact points of recombination and whether they represented one or two different recombination events.

As indicated above, the first bona-fide recombinant strain of HCV was obtained in St. Petersburg (Russia) in 2002 [26]. These authors identified an intergenotype recombinant between a subtype 2k and a subtype 1b. The recombination breakpoint was mapped to the NS2 gene, around position 3,175. Subsequently, the full genome sequence of this isolate was determined [28] and the initial breakpoint was confirmed. This led to the proposal of a new strain designation as RF1_2k/1b, in analogy to the nomenclature used for recombinant circulating forms of HIV. The authors identified two hairpin structures, denoted HS1 and HS2, in the vicinity of the proposed recombination breakpoint in the parental strains which were absent from the recombinant form. From this observation, they proposed a molecular mechanism for recombination in HCV consisting of template switching during the synthesis of the negative strand facilitated by the binding of HS2 to the RNA-dependent RNA polymerase which enabled 3′-terminal extension during recombination.

One important feature of this first report is that it documented not one but six different isolates, all derived from the same recombination event. This indicated that the recombinant strain was circulating in the population (all the isolates were obtained in a molecular epidemiology survey of HCV in St. Petersburg) and its origin was dated at least 10 years before the date of isolation. This same recombinant strain has been isolated in other countries (Ireland [29], Uzbekistan [30], Cyprus [31] and Georgia or France [32]) thus indicating that, even if it is not favored by natural selection, it is at least not selected against. This has important consequences for our understanding of the integration of different components of the HCV genome and proteome even after substantial divergence (the average genetic distance between different HCV genotypes is around 0.40 substitutions per site).

In addition to RF1_2k/1b, at least eight different intergenotypic recombinant forms (RFs) of HCV have been described and totally or partially characterized (Table 1). All HCV genotypes except for genotype 4 have been found in RFs and they have a wide geographic distribution. Interestingly, all RFs but one (RF_3a/1b, [33]) are formed by a 5′-end of genotype 2 and 3′-end of a different genotype, in which only subtype 1b appears in more than one RF. Given that some subtypes of genotype 2 and subtype 1b are usually found in older patients and not usually related to the relatively recent epidemic spread linked to increased usage of intravenous drugs [2], at present it is not possible to ascertain whether this pattern derives from adaptive features or is simply due to chance.

One seemingly common feature of intergenotypic RFs of HCV is that their breakpoints have been mapped to either gene NS2 or NS3, with a high proportion of them falling in a small range between amino acids 1,022 and 1,042. Although no detailed analysis of RNA secondary structure in the vicinity of this region has been reported so far, the small stretch of the HCV genome involved in recombination might be explained by a similar mechanism to that proposed for RF1_2k/1b [28], as detailed above.

HCV intersubtype divergence at the nucleotide level usually ranges between 0.2 and 0.3 substitutions/site. In consequence, the same procedures based on phylogenetic incongruence used to detect intergenotypic recombinants are applied to the detection of intragenotype and intersubtype RFs (Table 1). Until now, only five such cases have been described, four involving subtypes of genotype 1 and one involving subtypes of genotype 4. The geographic distribution of these RFs is also very wide and the same applies to the locations of the inferred breakpoints.

Interestingly, only two of the intersubtypic RFs have been fully sequenced [34,35] and, besides involving the same subtypes (1a and 1c), both include more than one breakpoint, resulting in mosaic patterns of recombination with portions corresponding to one subtype alternating with portions of another. Also, in both cases, these stretches are very dissimilar in size with one or a few relatively short segments of one subtype punctuating a genome of mostly the other subtype. There has been no detailed characterization of the nearby regions of the corresponding genomes and, as a result, no mechanism for the origin of these RFs has been put forward.

The other three cases of intersubtypic RFs have been characterized only partially at the genome level. One case was described recently by discordant phylogenetic assignment of the HCV E1- and NS5B-gene sequences obtained from an IDU patient from Portugal [36]. Simultaneous infection with two strains of different HCV subtypes was not fully discarded and no recombination breakpoint(s) has been proposed for this RF, which has to be considered only as a putative case awaiting further characterization. The two remaining cases of intersubtypic recombination have been described by the same research group [37,38] after sequencing one single stretch of the HCV genome in which the corresponding breakpoints were identified. The genes involved, core and NS5B, are relatively conserved and, in fact, are normally used for the phylogenetic typing and subtyping of HCV isolates. In consequence, a large number of sequences for these two regions are available in public databases, although only a small fraction of them were used in the corresponding analyses. In addition, the sequences used do not encompass the actual levels of genetic variation at the intrasubtype level of these regions. Insufficient or unbiased sampling of control sequences may provide artifactual support for recombinant strains, especially when only small regions and a few sequences are used to obtain the phylogenetic trees from which recombination is inferred.

Two additional reports of recombination in HCV have been published to date. Both involve intrapatient recombination of viruses circulating in individual patients undergoing therapy [44,45]. The first report partially analyzed a previously published data set [46] in which clone sequences were obtained at different times from the start of interferon+ribavirin therapy in search of escape mutations. HCV-RNA was isolated and RT-PCR products of the NS5A region were cloned and sequenced. In total, Moreno *et al*. analyzed 554 sequences that were sampled in weeks 0, 1, 2 and 4 from the start of the therapy in 6 of the 18 patients in the original paper by Puig-Basagoiti *et al.* [46]. The recombinant clone was found in a sustained responder patient and it corresponded to a sequence obtained in week 4 of therapy with putative parental sequences obtained in weeks 0 and 1 [44]. The recombination breakpoint was located in the PKR-binding region of ISDR (Interferon Sensitivity Determining Region), a portion in the NS5A putatively linked to sensitivity to interferon-based therapy (although this issue remains highly controversial). In this work, the authors reported in detail only the results of the positive case, in which apparently only four sequences were actually compared. Failure to consider the whole spectrum of variability in the population may create an artifactual view of recombination, as previously commented, and this possibility should be taken into account because further analyses reveal that other clones, with identical nucleotide sequences to the one identified as recombinant, were not identified as such.

Sentandreu *et al.* [45] analyzed over 17,700 sequences from two HCV genome regions, namely the E1–E2 region, encompassing hypervariable regions HVR1–HVR3, and a portion of NS5A, also including the ISDR. These sequences were obtained after cloning RT-PCR products of HCV-RNA obtained from 111 patients undergoing interferon+ribavirin therapy. No post-treatment sequences from sustained responders were available for study. The set of sequences from each patient was analyzed independently using the RDP3 software [47] and putative recombination events were considered only when at least three different methods concurred in identifying the same event. Further verification by maximum likelihood testing of each event led us to propose 43 recombination events from 17 different patients in the E1–E2 region, and 20 events from 9 patients in the NS5A region. The breakpoints proposed for each region were not distributed at random along the sequences but appeared to concentrate at specific areas. Although no structural analyses were performed in this study, these results are congruent with the involvement of RNA secondary structure in facilitating, if not determining, the hotspots or zones where recombination can occur within the HCV genome.

Finally, some additional cases of non-natural recombination in HCV have been published. For instance, Gao *et al.* [48] reported the detection of recombination in several experimentally-infected chimpanzees. Three chimpanzees were infected with HCV subtypes 1a and 1b simultaneously. The authors looked for HCV recombination by cloning and sequencing PCR fragments derived from the E1–E2 region. Recombination between subtypes 1a and 1b was found in two animals and they differed in the location of predicted breakpoint and order of subtype sequences. Recently, Reiter *et al.* [49] have analyzed the production of HCV recombinants in a replicon-based system with two marker mutations. Recombination in hepatic cell lines was necessary to restore the normal phenotype. In this experiment, recombination frequencies correlated with the distance between mutations and they were found to occur at a frequency of 4 × 10^−8^ per nucleotide.

## 3. Negative Reports of Recombination

Negative results are seldom published in scientific journals. Nevertheless, when they accumulate, they can be very informative and should be considered at least as indicators of an intrinsic difficulty in documenting the corresponding phenomenon. In consequence, before considering in more detail why it is apparently so difficult to document the existence of recombination in HCV, let us briefly consider a sample of negative reports after active searching.

Most negative reports of recombination in HCV are published as complementary analysis when a new HCV subtype is characterized. These analyses became practically the norm once the RF1_2k/1b was initially described [26,28]. For instance, Bernardin *et al.* [50] failed to detect any recombinant genome after fully sequencing HCV isolates from 3 HCV-double infected patients (two cases were 1a followed by a different 1a strain, and the others were 3a followed by 1a, 1a followed by 3a, and 1b followed by 1a). However, only one genome was analyzed from each patient, thus reducing the chances of detecting any recombination should there be.

Hmaied *et al.* [51] did not find evidence of recombination in the complete genomes of the two strains used to define subtype 4f. The same negative result was obtained by Bracho and coworkers in their analysis of subtype 1g [52] and another, yet unnamed, subtype of genotype 1 [53], by Martró *et al.* [54] in the description of subtype 2q, and by Gupte *et al.* [55] in their analysis of novel subtype 3i and an Indian variant of subtype 3a. Kuntzen *et al.* [56] did not find evidence of recombination when they first analyzed complete sequences of subtype 4k in addition to five new sequences of subtypes 4d and 4f.

Similarity analysis showed no evidence of inter- or intrasubtype recombination in the first complete genomes for subtypes 6c, 6l, 6o, 6p and 6q; corresponding to five HCV complete genome sequences from a blood donor in Thailand, and three Asian immigrants and one Caucasian in North America [57].

Viazov *et al.* [58] actively searched for recombination and coinfection with different HCV genotypes in two highly exposed populations of IDUs in Russia (n = 47) and Germany (n = 118). Only two cases of recombination, as deduced from discordant sequence-based subtyping in core and NS5B regions, were detected. Both corresponded to 2k/1b recombinants of Russian origin. Furthermore, only one case of mixed infection (a 1a/3a combination) was identified, which led the authors to conclude that the actual prevalence of recombinant HCV viruses and the necessary previous step of co-infection, at least with different genotypes, are very rare even in these highly exposed settings.

Magiorkinis *et al.* [59] performed a detailed analysis of full-length genome sequences of all HCV genotypes and subtypes available at that time. From their study, they concluded that there was no evidence for ancient recombination events with one possible, but yet unconfirmed, exception. This was derived from the analysis of genotype 4, for which one region (4195–4645) consistently inferred a different topology than the one proposed from the full length sequence, suggesting a strong monophyletic relationship with genotype 6. Given the ancient, close relationship between HCV genotypes 1 and 4, Magiorkinis *et al.* argue that the most probable reason for this observation is an ancient recombination event between ancestral strains of genotypes 1 and 6.

## 4. Why Is Recombination in HCV so Difficult to Detect?

The identification of several recombinant strains (Table 1) demonstrates that HCV is capable of successfully completing all the stages in the process: Simultaneous infection of the same cell by different viral strains, simultaneous replication of both viral genomes, template shift by the viral RNA polymerase while keeping the correct reading frame, and encapsidation and release of the recombinant genomes. The resulting products will then be subjected to the same population processes governing the maintenance, expansion or disappearance of new variants in a heterogeneous viral population. We have already indicated that at least RF1_2k/1b has been found circulating in different countries. If recombination in HCV is not impossible then, why is it so uncommon? We next consider several points that may, at least partially, explain why HCV recombination is so rarely detected.

One prerequisite for the production and detection of recombinant HCV is the simultaneous infection of the same cell by two parental viruses which differ sufficiently in their nucleotide sequences for a recombinant between them to be identified, usually through phylogenetic analysis. Previous reports [27] have shown superinfection exclusion (or homologous interference) in experimental cell cultures infected with HCV. This process implies that an HCV-infected cell becomes refractory to subsequent infection, thus severely limiting the possibility of recombination because the presence of two different viruses in the same cell after a secondary infection is prevented. However, the detection of intertype and intersubtype recombinant forms casts doubts on the validity of the previous conclusion, at least for natural infections, and indicates that simultaneous infection of the same cell by different HCVs, if not frequent, is at least possible, perhaps when the infection with the two strains is simultaneous. In fact, in cell-culture, simultaneous infection and co-replication of both viral genomes in the same cell has been detected, but when infections are performed sequentially, secondary infection is severely impaired [60].

A pre-requisite for simultaneous cell infection is that the individual is also infected by different viruses and this can be analyzed more easily than the more stringent condition of cell coinfection. Pham *et al.* [61] studied the prevalence of multiple infections (mixed infections, superinfections and reinfections) among IDUs in a prison setting and found by sequencing that 22 of 87 (25.3%) of the infected individuals with detectable HCV-RNA had two or more different virus strains. Lee *et al.* [33] analyzed the prevalence of double and triple infections among IDUs in different regions of Taiwan. They found that 14% and 1.3%, respectively, of 150 individuals analyzed carried viruses from two or three different HCV subtypes. Other reports on the prevalence of multiple HCV infection indicate values between 5% [62] and 39% [63]. Unfortunately, there are no reports on the frequency nor the type of cells simultaneously infected with two (or more) HCV subtypes *in vivo*, and the previous cases of coinfection at the organism level do not necessarily imply coinfection at the cellular level, although this is likely to occur given the previously described cases of recombination.

Most routine genotyping analyses of HCV are based on a single or only two small genome regions and do not require determining its nucleotide sequence [64]. Recombination in viruses can only be confirmed after a detailed and rigorous analysis of the patterns of variation found at the nucleotide level (see below), using good reference data sets and stringent statistical criteria that can differentiate clearly between a true recombination event and the accumulation of independent mutations that mimic the same final result expected after recombination. This is especially delicate and necessary for highly variable RNA viruses such as HCV.

Despite recent improvements in algorithms and computer power, the detection of recombination is often limited by computation. Some popular programs aimed at identifying recombination in nucleotide sequences are still rather limited in the amount of data they can process at a time. In consequence, researchers are often tempted to trim the data sets to be analyzed so they can be processed in reasonable times. However, this may lead to wrong conclusions because the reduction of data may create an artificial appearance of alternating similarity/dissimilarity patterns, subsequently assigned to different parental sequences, when in fact a more or less continuum spectrum of variants does exist. One such case is the likely reason for a report of intratype recombination involving subtypes 1a and 1b [38].

Alternatively, other authors have searched for methods with less demanding computational needs. For instance, Mes and van Doornum [65] have compared two different approaches for inferring recombination in the dominant and minority subtypes of HCV genotype 1. They used multiple alignments of partial or complete genome sequences, as available, and searched for signatures of recombination using the set of methods implemented in RDP3 [47] and the population genetic method developed by Betrán *et al.* [66]. The results obtained were markedly different with the two approaches. Although the phylogenetic methods implemented in RDP3 failed to detect any evidence for recombination, the population estimator of Betrán *et al.* revealed a large number of gene-conversion tracts. However, this result should be taken cautiously because Betrán *et al.*’s method was originally designed for detecting gene conversion in eukaryotes and its suitability and robustness when applied to rapidly varying genomes, such as those of RNA viruses like HCV, remains to be checked. The method relies heavily on the unlikelihood of simultaneous and independent occurrence of mutations, an assumption which is violated in HCV.

To sum up, although it is certain that HCV is able to fulfill all the required steps for the production of recombinant genomes with parentals of a wide range of divergence, it is also true that different barriers for their production and detection have been described. Their nature prevents us from obtaining a reliable estimate of the global frequency of recombination in HCV. Furthermore, it is not possible to evaluate whether the cases currently described are truly representative of the actual levels of recombination in this virus or if they represent the tip of a large, unappreciated iceberg.

## 5. Are Incongruent Trees Enough Evidence for Recombination?

Phylogenetic trees are an essential tool in the identification and characterization of recombination events in viruses. Although the term ‘recombination’ has become the standard for denoting the mechanism by which a genome with chimeric portions of separate genealogical or phylogenetic origin is formed, the actual process is closer to what geneticists usually describe as ‘gene conversion’. There are important differences at the molecular level between both processes but, in their results, the main difference is that gene conversion is asymmetric: usually only one offspring genome is a chimera of two parental genomes, which continue producing identical, clonal offspring sequences unless a separate “recombination event” takes place. In consequence, the identification of a recombinant genome requires that at least two portions of it can be traced back to different most recent common ancestors. This is only possible by phylogenetic reconstruction and, as expected, the specific procedures used for it play a key role in the validity of the conclusions. The ensuing considerations are valid not just for HCV but for most other viruses and, in general, organisms in which recombination has to be inferred from the different ancestry of portions of their genome.

Phylogenetic reconstruction is not an easy task, despite the recent development of user-friendly programs and packages for completing it. Many papers are published every year with a phylogenetic tree in which the authors are not aware of the intricacies and subtleties of the methods and choices they have applied, most often because they correspond to the default values in the programs or because they have followed the methods described in previous papers of similar contents or approaches. These are usually very bad choices and many published results would probably not hold once rigorous phylogenetic analyses are applied. There is only one possible way to work around this problem: to study seriously how and when to apply the different methods and to check that the data to be analyzed really fit the underlying, although often unstated, assumptions.

Our goal here is not to review the different methods and assumptions of phylogenetic reconstructions with viral sequences, for which several excellent resources are available. Instead, we will concentrate on a few specific points which are often skipped in the analysis of viral sequences, in general, and in the identification of HCV recombination, in particular.

Phylogenetic reconstruction is based on the analysis of shared character states resulting from common ancestry (homologies) and not from independent evolution (analogies). The first step in a phylogenetic analysis is, consequently, the establishment of the homology relationships among the different units (usually partial or complete genome sequences) to be compared. For nucleotide or amino acid sequences, this corresponds to their multiple alignment. Although multiple sequence alignments (MSAs) are usually straightforward for closely related sequences, hypervariable regions, especially those in which insertion/deletion events contribute significant variability, may be difficult to align. Occasionally it is preferable to remove poorly aligned portions (gblocks, [67]; trimal, [68]) than to derive wrong phylogenetic trees from an unreliable alignment. Protein coding regions should always be aligned using the derived amino acid sequences. There are two main reasons for this. First, it is this alignment which makes biological sense as indels introduced in the amino acid MSA will be ‘back-translated’ correctly into the corresponding nucleotide MSA. Tools for doing this are available even in popular phylogenetic analysis programs such as MEGA [69]. Secondly, the actual algorithms used for deriving the MSA work much more efficiently on a 20-letter than on a 4-letter alphabet, thus facilitating their task. There are a plethora of methods for MSA and interested readers should consult some of the excellent recent reviews on this topic to gain more information [70–72]. For untranslated regions (UTRs), the secondary structure of RNA should also be considered as an essential component of the homology relationships to be ascertained [73,74].

Once a satisfying MSA has been obtained, it is necessary to ascertain whether the data hold enough phylogenetic information to allow the reconstruction of their evolutionary history confidently. Lack of information can be due to an excess or to a defect of nucleotide substitutions. In the former case, too many changes may remain unaccounted for, even after applying sophisticated models of evolutionary change, and some sequences may enter into the “Felsenstein zone”, a region in which sequences appear to be similar because of the independent accumulation of identical changes (homoplasies) and not because of a shared evolutionary history, leading to the well-known artifact of “long-branch attraction”. This situation may arise when fast-evolving regions are used for phylogenetic reconstructions of sequences which shared a common ancestor a long time ago. In this case, the inferred phylogenetic tree does not correspond to the actual genealogical relationships of the corresponding sequences. A similar outcome can result when the number of nucleotide substitutions is very small, a typical situation in cases of very recent divergence from the common ancestor, especially when conserved or slowly-evolving genome regions are used in evolutionary inference. In these cases, a single homoplasy can artificially create groups of apparently shared ancestry but usually with a low bootstrap support. There are a few tools available for diagnosing these problems. One of the most popular methods is likelihood-mapping [75], a maximum likelihood implementation of quartet analysis [76] in which 4-taxa subsets of the MSA are used to compare the three possible unrooted trees. The total proportion of well-resolved quartets, those in which one tree is significantly better than the others, provides an estimate of the global phylogenetic signal in the MSA. When this analysis identifies a poor-signal MSA, no firm conclusions should be derived from it.

The next step in the phylogenetic analysis is to decide which method to use and, for those based on models of nucleotide or amino acid substitutions, to ascertain the best fitting model for the data. Although parsimony- and distance-based algorithms for phylogenetic reconstruction were favored for many years, recent developments in algorithms and computation power have allowed the popularization of maximum-likelihood and Bayesian-based approaches. The availability of more user-friendly software, although still requiring much more effort than simply “load the data and run”, is also helping to spread these methods which provide not only best evaluations of phylogenetic relationships for large datasets but also include a strong statistical background on which posterior analyses are based. As previously noted, this is not an adequate place to review this topic and the interested reader is referred to specific literature. Much the same can be said about the selection of evolutionary models. Fortunately, software developed by David Posada and his group allows for a relatively easy and straightforward evaluation of many of the most common, albeit simple, models for sequence evolution both at the nucleotide (jModeltest, [77]) and amino acid (ProtTest; [78]) levels. The relevance of using an appropriate evolutionary model in the inference of viral phylogenies was stressed by Posada & Crandall [79] and should be constantly taken into account.

Once these points have been considered, there is still one final step to take before accepting that two non-congruent phylogenetic trees represent a clear proof of a recombination event. Two different phylogenies may represent different phylogenetic histories that, for a certain dataset, do not differ (significantly) in statistical support, or they might be the result of analytical artifacts. The statistical support for the different phylogenies (which are nothing but different hypotheses about the evolution of the corresponding genome fragments) has to be evaluated and there are several methods for doing it. The most popular methods (SH, [80]; ELW, [81]) have been developed under a likelihood framework and involve the comparison of the likelihood of the different trees for each multiple alignment. The main factors which can lead to misleading tree inferences include (1) long-branch attraction, (2) composition bias of nucleotide and amino acid, and (3) convergent evolution. The two former factors are unlikely to operate within a species, but the third one should be considered cautiously in the analysis of RNA virus recombination.

## 6. Implications of Recombination in HCV

If there are not so many cases of recombination in HCV, why should we care about detecting and documenting them? There is, naturally, a genuine scientific interest in improving our knowledge of an important pathogen, from its evolution, epidemiology, natural history, replication, functional biology, *etc.*, all of which can be better understood from the analysis of natural and artificial recombinants. But a better knowledge of a pathogen should also be translated into practical applications.

We have previously mentioned that HCV is currently classified into several genotypes and subtypes. This classification is based on phylogenetic analysis of complete or partial genome sequences [8]. However, in clinical practice, HCV is usually typed by one of several commercial kits available which are aimed at detecting genotype- or subtype-specific mutations in conserved regions of the genome [64]. These typing tests target only one, or at the most two, small regions in the genome and, in consequence, cannot be used to detect recombinant strains. Furthermore, occasional mutations in those specific sites may lead to wrong typing of HCV strains. This is relevant from a clinical point of view because prognosis, natural history, and treatment recommendations as well as viral response may differ between HCV genotypes. Some genotypes, such as genotypes 2 and 3, are usually linked to better prognosis and response to standard interferon-based treatment, whereas others are linked to higher rates of treatment failure, such as genotypes 1 and 4. HCV genotype is currently included in the routine clinical evaluation for treating HCV chronic infection.

It is evident that recombinant strains may render at least some of these typing methods almost useless. In fact, Morel *et al.* [82] have recently advocated for new typing schemes for HCV based on at least two genome regions well apart in the viral genome (for instance, the 5′-UTR and NS5B gene). These tests should be carried out routinely as they would allow an easy identification of potential recombinants of different genotypes and even subtypes. As an additional precaution, these authors also propose the analysis of sequences of these or other similar regions when the expected response to therapy is not attained.

However, a new era of therapeutics against HCV is just starting and the new antivirals are targeted against specific regions or positions of the HCV genome or proteome. Just as in many other RNA viruses, natural resistance variants in the HCV genome that allow the virus to avoid the effects of these drugs are already known [83,84] and the widespread use of these new treatments will eventually increase the number and frequency of resistance mutations. One of the basic strategies for preventing the spread of these variants and ultimately attaining the goal of eliminating or at least controlling the infecting virus below certain thresholds, is to simultaneously treat with drugs targeted to different proteins. The aim is to reduce the chances of resistance mutations simultaneously arising in the same individual genome that allow the corresponding viral particle to spread because its offspring can survive the antiviral drug selection. In these cases, recombination can act as a catalyst for antiviral resistance because it is no longer necessary that the two escape mutations arise simultaneously in the same genome; they can be brought together as a result of recombination between the appropriate parentals. Consequently, a more intense surveillance of recombination in HCV is necessary to accommodate future therapeutics to prevent, or at least limit, the appearance and spread of resistance mutations.

A final implication of HCV recombination is the relevance from epidemiological and public health perspectives. In most countries, hepatitis C is a disease of mandatory communication to public health authorities. Given the high transmissibility of this virus and the important personal and social consequences of its infection, many countries have implemented an active surveillance of the main circulating genotypes and subtypes of HCV. This information is very valuable from an epidemiological point of view and recombination, for the same reasons indicated above, can mislead the basic information on the virus types found in a given population.

## 7. Conclusions

Recombination in HCV is still a rare event and the number of well-documented cases is still very low. There are difficulties at different levels for the production and detection of recombinant strains of this virus and, although there is still much uncertainty about its actual incidence, it is likely that the levels of recombination in HCV are being underestimated. This may have important practical consequences in the clinical and epidemiological settings, especially with the introduction of new therapeutic drugs for which specific resistance mutations are already known. Recombination can eventually facilitate combination of single resistance mutations in the same genome, and active epidemiological surveillance of recombinant forms will be of public health interest.

## Figures and Tables

**Table 1.** Summary and main features of the published cases of recombination in Hepatitis C virus (HCV). The phylogenetic analyses column summarizes the method used for inferring the phylogenetic trees (NJ: neighbor-joining, ML: maximum likelihood), along with the evolutionary model (K2P: Kimura 2 parameter, TN: Tamura-Nei; GTR: general time reversible, G: gamma distribution for heterogeneity among sites), method for evaluating branch support (boot: bootstrap resampling with number of pseudorandom replicates, aLRT: approximate likelihood ratio test), and method or software used for testing and/or identifying recombination breakpoints.

**Strain**	**Genotype**	**Country**	**Recombination breakpoint(s)**	**Phylogenetic analyses**	**Refs.**
**Intergenotype**					
RFl_2k/1b	2k/1b	Russia, Ireland, Uzbekistan, Georgia/France, Cyprus	NS2, positions 3175–3176	NJ-??-?boot; Simplot, Bootscan	[26,29–32]
D3	2i/6p	Vietnam	NS2/NS3 junction, between positions 3405 and 3464	NJ-K2P-1000boot; Simplot, Bootscan	[39]
SE-03-07-1689	RF3_2b/1b	Philippines	NS3, positions 3466–3467	NJ-??-?boot (CLUSTALW); Similarity plot (RAT)	[40]
Rl	2/5	France	NS2/NS3 junction, between residues 3420 and 3440	NJ-TN+G-100boot(MEGA); RDP	[41]
D177	RF_2b/6w	Taiwan	NS2/NS3 junction, position 3429	NJ-K2P-1000boot,Simplot	[33]
	RF_3a/1b	Taiwan	undetermined	NJ-K2P-1000boot,Simplot	[33]
	RF_2a/1a	Taiwan	undetermined	NJ-K2P-1000boot,Simplot	[33]
HC10-0804	2b/1b	Japan	NS2/NS3 junction, positions 3443–3444	NJ-K2P-1000boot,Simplot and Bootscan	[42]
JF779679	2b/1a	USA	NS2/NS3 junction, positions 3405–3416	NJ-??-?boot (CLUSTALW); Simplot	[43]
**Intersubtype**	Subtypes				
PE22	RF2_1b/1a	Peru	NS5B, position 8321	NJ-K2P-1000boot (MEGA); Simplot, LARD	[37]
HC-J1	1a/1c	Japan	2 sites in E1–E2, at positions 1407 and 2050	No PhylTree; Simplot, Bootscan	[34]
Khajal	1a/1c	India	5 sites, from core to NS3, at positions 801, 1261, 2181, 3041, and 3781	ML(Modeltest), 5000 boot (NJ); Simplot, Bootscan	[35]
H23	1b/1a	Uruguay	core, at position 387	ML-GTR+G, aLRT (Phyml); GARD, LARD	[38]
R49	4a/4d	Portugal	undetermined	NJ-K2P-1000boot	[36]
**Intrapatient**	Subtype				
	1b	Spain	NS5B, at the residue 286	No PhylTree; Simplot, Bootscan	[44]
	1a, 1b, 3a	Spain	1 or 2 sites within E1E2 or NS5A	ML, GTR+G, 1000boot (Phyml); RDP3 (at least 3+); SH + ELW (TreePuzzle)	[45]
